# A randomized trial to optimize HIV/TB care in South Africa: design of the Sizanani trial

**DOI:** 10.1186/1471-2334-13-390

**Published:** 2013-08-26

**Authors:** Ingrid V Bassett, Janet Giddy, Christine E Chaisson, Douglas Ross, Laura M Bogart, Sharon M Coleman, Tessa Govender, Marion Robine, Alison Erlwanger, Kenneth A Freedberg, Jeffrey N Katz, Rochelle P Walensky, Elena Losina

**Affiliations:** 1 Division of General Medicine, Massachusetts General Hospital, Boston, MA, USA; 2 Division of Infectious Diseases, Massachusetts General Hospital, Boston, MA, USA; 3 Medical Practice Evaluation Center, Massachusetts General Hospital, Boston, MA, USA; 4 Harvard Medical School, Boston, MA, USA; 5 Harvard University Center for AIDS Research, Harvard University, Cambridge, MA, USA; 6 McCord Hospital, Durban, South Africa; 7 Data Coordinating Center, Boston University School of Public Health, Boston, MA, USA; 8 St. Mary’s Hospital, Mariannhill, Durban, South Africa; 9 Division of General Pediatrics, Department of Medicine, Boston, Children’s Hospital, Boston, MA, USA; 10 Department of Epidemiology and Biostatistics, Boston University School of Public Health, Boston, MA, USA; 11 Department of Health Policy and Management, Harvard School of Public Health, Boston, MA, USA; 12 Department of Orthopedic Surgery, Brigham and Women’s Hospital, Boston, MA, USA; 13 Division of Rheumatology, Brigham and Women’s Hospital, Boston, MA, USA; 14 Division of Infectious Disease, Brigham and Women’s Hospital, Boston, MA, USA; 15 Department of Biostatistics, Boston University School of Public Health, Boston MA, USA

**Keywords:** (3-10): HIV/AIDS, Tuberculosis, South Africa, Linkage to care, SMS reminders, Randomized controlled trial, Counseling/Support

## Abstract

**Background:**

Despite increases in HIV testing, only a fraction of people newly diagnosed with HIV infection enter the care system and initiate antiretroviral therapy (ART) in South Africa. We report on the design and initial enrollment of a randomized trial of a health system navigator intervention to improve linkage to HIV care and TB treatment completion in Durban, South Africa.

**Methods/Design:**

We employed a multi-site randomized controlled trial design. Patients at 4 outpatient sites were enrolled prior to HIV testing. For all HIV-infected participants, routine TB screening with sputum for mycobacterial smear and culture were collected. HIV-infected participants were randomized to receive the health system navigator intervention or usual care. Participants in the navigator arm underwent a baseline interview using a strengths-based case management approach to assist in identifying barriers to entering care and devising solutions to best cope with perceived barriers. Over 4 months, participants in the navigator arm received scheduled phone and text messages. The primary outcome of the study is linkage and retention in care, assessed 9 months after enrollment. For ART-eligible participants without TB, the primary outcome is 3 months on ART as documented in the medical record; participants co-infected with TB are also eligible to meet the primary outcome of completion of 6 months of TB treatment, as documented by the TB clinic. Secondary outcomes include mortality, receipt of CD4 count and TB test results, and repeat CD4 counts for those not ART-eligible at baseline. We hypothesize that a health system navigator can help identify and positively affect modifiable patient factors, including self-efficacy and social support, that in turn can improve linkage to and retention in HIV and TB care.

**Discussion:**

We are currently evaluating the clinical impact of a novel health system navigator intervention to promote entry to and retention in HIV and TB care for people newly diagnosed with HIV. The details of this study protocol will inform clinicians, investigators, and policy makers of strategies to best support HIV-infected patients in resource-limited settings.

**Trial registration:**

Clinicaltrials.gov. unique identifier: NCT01188941.

## Background

The dual epidemics of HIV and TB remain a leading health care challenge in South Africa [1,2]. Despite rapid expansion in the availability of antiretroviral therapy (ART), only about half of HIV-infected individuals are in care [3]. The period following HIV diagnosis but prior to ART initiation represents a time of very high mortality, attributable in part to late TB diagnosis and poor rates of TB treatment completion [2,4-6]; TB remains the leading cause of death among HIV-infected individuals [7]. Interventions focused on integrating intensive TB screening into HIV diagnostic services and improving linkage to HIV and TB care would be of substantial benefit in controlling these epidemics.

Previous studies have shown that the paucity of symptoms, transportation barriers, stigma, and lack of self-efficacy and social support are all factors that negatively affect the likelihood of entering and remaining in HIV care [8,9]. While most interventions have focused on retaining patients in care or improving ART adherence [10-14], few studies have addressed the multiple barriers faced by HIV-infected individuals before entering care. A randomized controlled trial of a brief case manager intervention in the US improved linkage and retention in care at 12 months [15,16]. However, no randomized studies of case managers or navigators to improve linkage to HIV care have been reported from resource-limited settings with high HIV and TB prevalence.

This paper describes the rationale and the design of a randomized controlled trial that aims to establish the efficacy of an in-person and mobile phone-based health system navigator (HSN) intervention in improving linkage to HIV and TB care among newly diagnosed HIV-infected outpatients in Durban, South Africa, and to evaluate the cost and cost-effectiveness of this intervention. We hypothesize that the HSN intervention will improve linkage to and retention in care for these diseases and lead to higher rates of ART initiation and TB treatment completion compared to the standard of care.

## Methods

### Study rationale

The design of this study is grounded in the Andersen model of health services utilization, which identifies environmental and patient characteristics that influence health-seeking behaviors [17,18]. Andersen recognizes predisposing factors (i.e. demographic characteristics), enabling factors (factors that represent the actual ability to obtain care), and perceived needs (symptoms, values, and knowledge about health and the health care system), which together determine an individual’s use of the health care system [17,18]. The goal of the trial is to evaluate the efficacy of a strengths-based case management approach, implemented by HSNs, for effecting modifiable patient factors that will improve linkage to HIV and TB care [19].

### Study design

**Figure 1:**
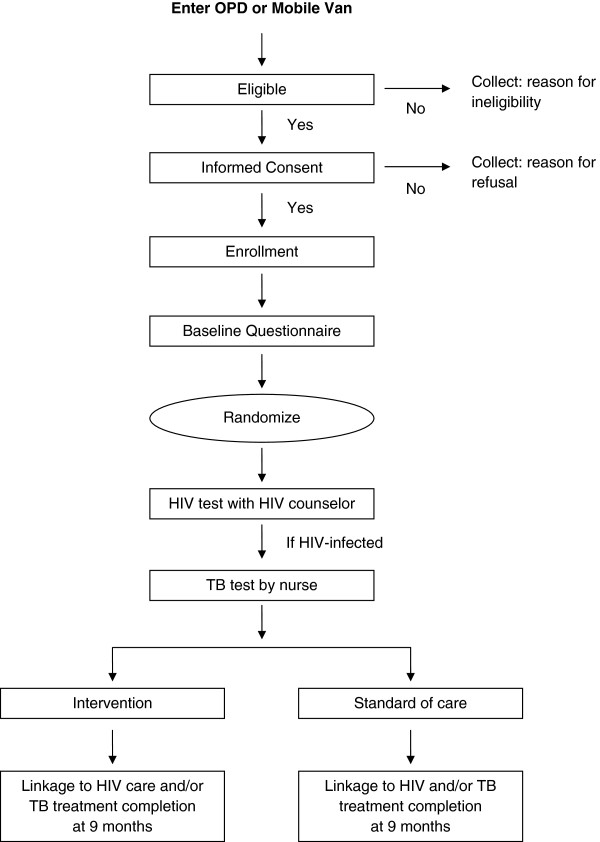
**Consort diagram of the study design.** OPD: outpatient department.

The study is a multi-site randomized controlled trial, known locally as the Sizanani (Zulu for “Help Each Other”) Trial. Participants were enrolled at four sites in the greater Durban area. These sites comprised two hospital-affiliated outpatient departments and two municipal clinics (e.g. nurse-driven primary health care sites). All patients were enrolled in the study and randomized prior to HIV testing to reduce differential acceptance rates by results of HIV testing. Participants were tested for HIV and all newly-identified HIV-infected participants received TB screening. HIV-infected participants were either in the intervention group, and were assigned to a HSN, or the control group, the current usual care in Durban (Figure 1). Nine months following enrollment, medical records will be reviewed, and all participants will be contacted by telephone to assess linkage to HIV care and TB treatment completion. Follow-up for the study is ongoing.

### Randomization

After completing the baseline questionnaire, enrolled participants were randomized, prior to HIV testing, to the intervention or the usual care arm. Randomization was stratified by site and gender, with blocks of varying length. Randomization assignments were created by the Boston-based Data Coordinating Center. A randomization table that was inaccessible to the South African research assistants was embedded in a Handheld Assisted Patient Interview (HAPI) device and provided randomization assignments for consecutive study numbers.

### Setting and participants

Participants were enrolled at four outpatient sites in KwaZulu-Natal. McCord Hospital predominantly serves an urban population in the greater Durban area. McCord was part of the PEPfAR-funded scale-up of ART from 2004 to 2012. St Mary’s Hospital is a state-aided district hospital, serving a peri-urban population, which began offering PEPfAR-funded ART starting in 2003. In addition, participants were enrolled in two primary health municipal clinics: Mariannridge and Tshelimnyama. In August 2012, the McCord enrollment site closed; enrollment continued at the other three sites. All adults (≥18 years old), English- or Zulu-speaking were eligible if they met the following criteria: presenting Monday through Friday for outpatient care, voluntarily had an HIV test, were able and willing to give informed consent, reported no prior positive HIV test, were willing to share HIV and TB test results with research staff, and were not known to be pregnant at the time of screening.

### Enrollment procedures

Eligible participants signed a consent form (in English or in Zulu) in a private area, and the research assistant then administered a baseline questionnaire comprised of detailed demographic information, as well as clinical information including depression, social supports and self-identified barriers to engaging in care. At enrollment, participants also provided contact details, as well as the contact information for a family member or friend who could be telephoned if the participant could not be reached. Participants were asked their preference for Zulu or English SMS text. Participants then underwent counseling and rapid HIV testing by the study sites’ HIV counseling staff as per standard South African protocol [20] and received test results immediately. All HIV-infected participants were counseled regarding the meaning of the HIV test result, the appropriate next steps, and how to obtain HIV treatment if needed; they were offered venipuncture for a CD4 count, and instructed to return for results approximately two weeks later. HIV-infected participants were then seen by a Sizanani nurse who administered a brief standardized TB symptom questionnaire and obtained history related to prior and current TB treatment. Participants expectorated a single sputum specimen, spontaneously or using nebulized 3% hypertonic saline, if needed. Sputum specimens were transported daily to the TB laboratory of the University of KwaZulu-Natal. All sputum samples were stained to assess for acid fast bacillus (AFB) smear, and were processed for *Mycobacterium tuberculosis* culture with drug susceptibility testing. The TB nurse contacted participants upon receipt of a positive AFB or culture, and they were directed to the site TB nurse for registration and TB treatment initiation.

### Health System Navigator (HSN) intervention

**Figure 2:**
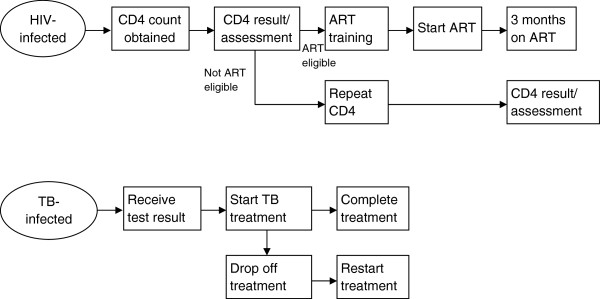
**HIV and TB care pathways.** ART: Antiretroviral therapy; TB: tuberculosis.

The HSN provided focused personalized support to patients in the period immediately following HIV diagnosis, with or without an accompanying TB diagnosis, to improve linkage to care. The HSN sought to identify and influence modifiable patient factors, including self-efficacy and social support, through a strengths-based case management approach [19,21]. The HSN conducted a brief strengths-based interview to help patients identify their own assets to overcome barriers to obtaining care. They were also provided with lists of local HIV and TB care facilities to assist participants with identifying a convenient and acceptable care site if the participant did not want to receive care at the study site. The HSNs provided their study mobile phone number to participants, and were available to respond to questions. Lastly, the HSNs physically showed participants the location where blood could be taken for a CD4 count, as well as the location of the HIV clinic. Participants then continued with their routine care at the study site. Once the patient left the clinic, the HSN contacted the subject through regularly scheduled SMS reminders to retrieve test results and keep appointments, as well as providing psychosocial support through regular telephone calls (Table 1). During each telephone contact, the HSN assessed where participants are on the HIV and TB care pathway (Figure 2). The HSN downloaded weekly reports from the study database which contains lists of patients due for SMS and telephone calls. Each SMS was delivered in the subject’s preferred language and did not mention the study site, HIV or TB. The content of the SMS was standardized, but the message was dictated by the subject’s position in the care pathway. For example, if blood for a CD4 count had been taken, but the subject had not yet picked up the results, the SMS read “Sizanani: please pick up results in the next week.” All patient contacts and the time required for attempted patient contacts were logged daily by the HSN to allow for an assessment of the personnel time required to perform the intervention.

**Table 1:** Health System Navigator (HSN) phone call and SMS schedule

**Week Number Post-Enrollment**									
		**1**	**1.5**	**3**	**4**	**6**	**8**	**12**	**16**
Type of Contact*	**SMS**		X	X		X		X	
	**Phone**	X			X^†^		X	X	X‡

^*^If participants are not reached at the time of a telephone call, the contact schedule is intensified by turning the next designated SMS reminder into a phone call. For example, if a subject is not reached at the week 1 phone call, the week 1.5 SMS becomes a phone call. The contact schedule differs slightly by study site, based on the site-specific time to return for CD4 count results.

^†^HSN calls subject’s alternative contacts if the subject is still unreachable.

^‡^ Participants are considered unreachable if the HSN still cannot contact them.

### Usual care

Patients randomized to the usual care arm were given a date and time to return two weeks later to receive their CD4 count results from the HIV counselors. As in the intervention arm, patients with a positive HIV test were redirected to the TB focal point at each site. A trained study nurse administered a brief questionnaire regarding TB symptoms and collects a sputum specimen. Patients then proceeded to the clinician, where they resumed their usual care. The participants in the usual care arm had no further contact with the study staff until the 9-month follow-up phone call.

### Research personnel training

The study staff included a project manager, one enrolling research assistant at each site, one HSN at each site, one TB nurse at each site, two follow-up research assistants, and two data entry personnel. Training was conducted by a team consisting of the principal investigator, a behavioral scientist, a Durban-based social worker, and the head of the Data Coordinating Center, and included lectures, question/answer, information-sharing, interactive problem solving and role plays. Some training was common to all study staff. TB nurses received training in universal precautions and safe handling of sputum specimens. The HSNs, experienced counselors with more advanced education, received training in strengths-based case management [19]. HSNs were provided with standardized definitions of patient barriers (for example, competing needs, transportation, fear) and strengths (for example, self-efficacy, familial emotional support, spirituality) assessed at each patient contact. In addition, HSNs were given scripts and probes to prepare for their encounters with patients. The staff also received training in the ethical conduct of research and confidential handling of data from the principal investigator and the NIH online course on the protection of human research participants [22].

### Follow-up process

Outcomes regarding successful linkage to each step on the HIV and TB care path are ascertained through review of medical records and registers at the study sites. In addition, external sources are used, including TB outcomes from the Department of Health as well as deaths as ascertained through the South African death registry. Finally, all participants, regardless of study arm, are contacted by telephone for a follow-up questionnaire, with a minimum of 3 call attempts starting at 9 months and finishing 12 months post-enrollment. The purpose of the 9 month follow-up questionnaire is to ascertain information regarding HIV care obtained outside of the study sites, as well as changes in family composition, emotional health, social support, and barriers to care compared to enrollment.

### Outcome measures

#### Primary outcomes

The primary outcome of the study is linkage to and retention in care assessed 9 months after enrollment for those HIV-infected individuals eligible for ART or TB treatment. The primary outcome is defined as: 1) 3 months on ART for ART-eligible HIV-infected patients, and 2) 6 months of TB treatment or 3 months on ART for HIV-infected patients co-infected with TB. Thus, for co-infected patients, a patient who links to either HIV or TB treatment is considered to have successfully reached the primary outcome. In the primary analysis, we will use data obtained from medical record review and TB treatment outcomes as documented by the Department of Health to minimize ascertainment bias in the navigator arm. HIV care received outside of the study sites will be assessed through self-report and will be used in sensitivity analyses.

#### Secondary outcomes

HIV-infected participants who are not eligible for ART or TB treatment at baseline cannot reach a primary outcome, but may reach a secondary outcome. We will examine mortality at 9 months, receipt of CD4 count results, receipt of TB laboratory results, attending ART literacy training for eligible participants, and repeat CD4 count for ART-ineligible participants at 6 months after enrollment as secondary outcomes. We hypothesize that people in the intervention group will be more likely to undergo CD4 count testing and retrieve their CD4 count and TB results.

Secondary outcomes will be derived from data collected via medical record review and participant 9-month follow-up interviews. Family members who answer a deceased subject’s telephone will be queried regarding date and cause of death, if known. A list of reported deaths and all unreachable patients will be cross-referenced with the South African death registry using South African identification numbers. Participants will be considered lost to follow-up if they or their family were unreachable by the study staff and do not appear in the national death registry.

### Sample size

Based on our prior work at McCord and St. Mary’s Hospitals, we estimated the HIV prevalence to be 35% among enrollees, with approximately 20% of HIV-infected enrollees co-infected with TB [23-25]. Based on the ART-eligibility criteria at the start of the study, we anticipated that 22% of enrolled participants would be eligible to meet a primary outcome. Based on our prior work, we expected 35% of HIV infected/TB negative patients in the control group to achieve the primary outcome. Our power considerations were based on the assumption that the intervention will affect HIV and TB treatment outcomes similarly, which we thought reasonable given the lack of data on linkage to care interventions that distinguish between these groups. The study was originally powered (90%) to detect a 27% increase in linkage to care in the intervention arm. We planned to enroll 4,745 participants in total, with 1,661 HIV-infected individuals eligible to reach the study outcome.

In June 2012, the McCord Hospital HIV clinic closed due to the conclusion of PEPfAR funding, thus we ceased enrollment at the McCord outpatient department because participants could no longer meet the primary outcome at the enrollment site. We redeployed the McCord team to Mariannridge Clinic beginning in August 2012. Additionally, with the approval of the trial DSMB, we used actual trial data on HIV prevalence and the proportion who receive a CD4 count to refine our sample size requirements. The updated sample size called for an additional 149 patients, for a new total of 4,894 participants, 1,714 of whom are HIV-infected.

### Data collection

For HIV-related outcomes, the following data elements are collected: dates and results of HIV test, CD4 count and viral loads if available, dates of ART training, and ART initiation. Data are obtained from electronic and paper records, as well as patients’ self-report (Table 2). Data on mortality will be obtained from clinical records, follow-up telephone contacts, and the national death registry.

**Table 2:** List of study instruments, data elements and source of information

**Data collection instruments**	**Time of ascertainment**	**Data collected**	**Source**
Pre-screen checkpoints	Day of enrollment	Basic demographic information and eligibility assessment	Subject self report
Screening and baseline questionnaire	Day of enrollment	Demographic information, emotional health, social support, barriers and competing needs to care	Subject self-report
Contact information	Day of enrollment	Contact information to reach subject, including preferred language for SMS	Subject self-report
TB nurse questionnaire	Day of enrollment	TB symptoms and TB treatment history	Subject self-report
Health system navigator contact log	Day of enrollment and throughout	Perceived barriers to care and strengths to overcome barriers, time spent contacting participants	Medical record, subject self-report
Participant log	Day of enrollment and throughout	HIV test and CD4 results and retrieval, ART initiation, TB treatment dates and outcomes, date last seen	Medical record, death registry, subject self-report
9-month follow-up form	Patient telephone interview 9-mo after enrollment	HIV and TB care, demographic and clinical updates, emotional health, social support, and competing needs	Subject self-report
Study completion form	At 9-mo follow-up	Study endpoint (death, loss to follow-up, completed follow-up)	Medical record, HSN log, death registry

TB: Tuberculosis; HSN: Health system navigator.

For TB outcomes, the site of TB treatment, date of TB treatment initiation, date of TB treatment completion, date of assigned treatment outcome, and treatment outcome (cured, treatment completed, treatment defaulted, treatment failure, death) will be collected from study site TB registers as well as from the Department of Health’s TB outcomes database.

Information on research personnel resource utilization will be collected, such as the HSN time spent in face-to face contact with patients, sending SMS, and telephoning participants. This will be used to inform an anticipated cost-effectiveness analysis. Table 2 provides a complete list of data collection tools.

### Analysis plan—primary and secondary outcomes

The primary analysis will use the intent to treat approach. All randomized participants eligible to meet a study outcome will be analyzed according to the arm they were assigned to evaluate the efficacy of the navigator intervention. This analysis will include those whose outcome cannot be ascertained at follow-up. We will assume conservatively that participants who cannot be found by study personnel and who do not have data available in the medical record were not linked to care.

The first phase of the analysis will evaluate the success of randomization in distributing baseline characteristics evenly between the intervention and the usual care arms. Baseline characteristics that are not balanced across the study arms will be advanced into regression models, evaluating the efficacy of the intervention, to address possible confounding. Characteristics that are balanced but that have an association with the outcome will also be advanced to final models to reduce error variance. To evaluate the hypothesis that the intervention will improve linkage rates, we will use adjusted logistic regression modeling to determine successful linkage to care. Secondary outcomes will be analyzed in a similar fashion.

We will conduct a series of hypothesis-generating secondary analyses to examine the potential impact of several predisposing (age, gender), enabling (social support, self-efficacy), and perceived need factors (reason for HIV testing) from the Andersen model [17,18] as moderators and mediators of the intervention. We will build several logistic regression models, one at a time, with interaction between study arm and age, gender, social support scale and reason for HIV testing, due to capped sample size. These analyses will demonstrate whether the intervention is differentially more efficacious in particular patient subgroups. If the intervention is efficacious in improving linkage to care, we will perform additional analyses that attempt to identify the mechanism of intervention effect, for example, whether the intervention improves social support and self-efficacy, and whether these improvements enhance linkage to care. This hypothesis-generating analysis will be valuable for determining whether future studies of this intervention should target particular patient subgroups and whether certain effects of the intervention (e.g. improvement in self-efficacy) are especially integral to intervention efficacy.

### Ethics

Prior to trial initiation, investigators obtained approval for all study components from the McCord Hospital Medical Research Ethics Committee, the St. Mary’s Hospital Research Ethics Committee and the Partners Institutional Review Board (Protocol 2011-P-001195, Boston, MA). After the closure of the McCord study site, the McCord Hospital Medical Research Ethics Committee transferred the protocol to the University of KwaZulu-Natal Biomedical Research Ethics Committee (BREC). Questionnaires and consent forms were created in English, translated into Zulu, and back-translated; all translations and back-translations were submitted for Ethics Committee review. Potential risks of participation in this study are minimal to moderate. The risk of stress and anxiety due to discussion about HIV, HIV testing, and disclosure of HIV status is minimized by conducting all discussions in a private space. Recruitment prior to HIV testing also reduced the burden on the participant. There is a risk of breach of confidentiality due to shared cell phone use. To reduce this risk, SMS intentionally contain no patient identifiers, and do not refer specifically to HIV, TB, or the study site. None of the information collected through this investigation will likely affect a subject’s relationship with other individuals (e.g., patient-physician, family relationships) or influence the subject’s HIV treatment at the research site. All data are protected in locked cabinets or in password-protected computers equipped with anti-virus software.

## Enrollment results

### Enrollment

Enrollment began on August 11, 2010 and ended on January 16, 2013. Of 6,536 individuals screened, 4,954 (76%) met eligibility criteria; of those 4,903 (99%) were enrolled over 29 months. In total, 1,546 people were enrolled at McCord (enrollment stopped at this site on August 6, 2012), 2,188 at St Mary’s, and 1,169 at the two primary health clinics. Overall, 39% of the participants enrolled were HIV-infected (range: 30%–51%, depending on the enrollment site), for a total of 1,899 HIV-infected participants. As of July 15, 2013, 1,680 participants had available TB sputum culture results and 216 had no sputum sample available. Of those with culture results, 377 (22%) were identified as actively TB-infected by smear and/or culture.

### Follow-up

Follow-up and outcome ascertainment is ongoing, and remains blinded to follow-up rates and outcomes across arms. As of July 15, 2013, 114 participants (6%) had not yet reached their 9-month assessment; 52 participants (3%) were due for their 9-month follow-up. 1,329 (70%) participants had completed their 9-month; 252 participants (13%) had died or withdrawn. Overall, this yields a 90% follow-up rate (87-90%) at the current time.

## Discussion

Innovative strategies are urgently needed to improve linkage to TB and HIV care, so that South Africans can reap the maximum benefits from currently available life-saving therapies. Although HIV testing efforts have expanded recently in South Africa [26], a substantial proportion of people newly diagnosed with HIV are not retained in the care system [24,27-30]. While evidence supports that behavioral and cognitive interventions can be effective for improving ART adherence in sub-Saharan Africa [31,32], few studies have rigorously evaluated approaches for increasing linkage to HIV care in high-prevalence regions [31].

The Sizanani Trial is unique in its well-articulated conceptual framework and its focus on the period immediately following HIV diagnosis. The Sizanani intervention includes an initial in-person strengths-based case management session with a HSN, followed by both SMS reminders and individualized phone support [15,16,19]. Phone calls and SMS reminders are tailored to each participant’s stage on the HIV or TB care pathway, so that content is maximally relevant. The study focuses on improving both HIV and TB outcomes, given the deadly synergy between these diseases [33,34]. Participants are enrolled prior to their HIV test to reduce differential acceptance rates by results of HIV testing, facilitating a representative sample of newly diagnosed individuals. The study enrollment rate is very high (99%), similar to a study of SMS reminders for ART adherence in Kenya [10], which may reflect participants’ perception of improved access to care through participation in research [35].

Understanding better ways of linking and retaining HIV-infected people in care has become increasingly urgent in light of changing priorities in HIV treatment and prevention initiatives. Test and treat programs cannot be successful without high rates of linkage to care [36,37], the value of testing is substantively reduced if newly diagnosed individuals do not successfully initiate ART to improve their own health. Similarly, retaining patients in care and rapidly initiating them onto treatment is crucial for decreasing HIV transmission for “treatment as prevention” [31,38,39]. If the health system navigator intervention is efficacious, this may represent an important adjunct to biomedical interventions for decreasing secondary transmissions. One mathematical simulation study of HIV testing and treatment strategies in South Africa found that improving linkage to and retention in care was associated with a 55% lower rate of new infections compared to universal testing and treatment alone [36]. If the HSN is effective at improving linkage to HIV and/or TB care, we will be uniquely poised to evaluate the cost, cost-effectiveness, and long-term clinical impact of the intervention.

This design has several limitations. We assume that the majority of participants link to care at the four study sites where medical records will be reviewed. However, participants could enter care at other clinics offering HIV care in Durban. This problem was exacerbated when the McCord enrollment site closed, and participants enrolled there were required to seek HIV care elsewhere. We are actively evaluating their outcomes. Specifically, we are determining how many McCord HIV clinic patients were successfully transferred to another clinic [40]. In addition, the use of self-report introduces some reporting bias; however, the primary analysis will be based on outcomes obtained using medical records, clinic registers, TB outcomes reported to the Department of Health, and the national death registry.

Despite the increasing availability of ART for HIV-infected individuals in South Africa, patients continue to access care very late and suffer from extremely high rates of mortality early after diagnosis. A substantial proportion of those diagnosed with HIV never link to HIV care and therefore fail to gain access to the personal and public health benefits of HIV and TB treatment. Yet little is known about how best to target patient- and contextual-level interventions to improve access to care. This trial has the potential to inform physicians, government, and policy makers on how to maximize the benefits of both ART and TB treatments through a strategy of timely and integrated HIV and TB diagnosis and linkage to care.

## Competing interests

The authors declare that they have no competing interests.

## Authors’ contributions

IVB, CEC, RPW, KAF, EL, DR, JG, and JNK conceived and designed the study. IVB, TG, SMC, and JG collected and assembled the data. IVB and MR drafted the article. CEC, LMB, DR, JG, SMC, TG, AE, KAF, JNK, RPW, and EL critically revised the article for important intellectual content. All authors read and approved the final manuscript.

## Pre-publication history

The pre-publication history for this paper can be accessed here:

http://www.biomedcentral.com/1471-2334/13/390/prepub
